# The prevalence of rheumatic heart disease in Ethiopia: a systematic review and meta-analysis

**DOI:** 10.1186/s40794-023-00192-y

**Published:** 2023-10-13

**Authors:** Hiwot Berhanu, Yimer Mekonnen, Abdulhalik Workicho, Kalkidan Hassen, Zenebe Negeri, Morankar Sudhakar, Shimelis Mitiku, Andualem Mossie

**Affiliations:** 1 Department of Biomedical Sciences, Faculty of Medical Science, Jimma Institute of Health, Jimma University, Jimma, Ethiopia; 2 Department of Pharmacy, Faculty of Health Sciences, Jimma Institute of Health, Jimma University, Jimma, Ethiopia; 3 Department of Epidemiology, Institute of Health, Jimma University, P.O.Box 378, Jimma, Ethiopia; 4 Department of Nutrition and Dietetics, Institute of Health, Jimma University, Jimma, Ethiopia; 5 Department of Health, Behavior and Society, Faculty of Public Health, Ethiopian Evidence Based Health Care CenterInstitute of HealthJimma University, Jimma, Ethiopia

**Keywords:** Rheumatic heart disease, Prevalence, Echocardiography, Ethiopia

## Abstract

Globally, more than 33 million people are living with rheumatic heart disease (RHD). A high prevalence of the disease is observed in people with poor socio-economic status, overcrowding, and low access to medical facilities. Even though different studies have been conducted in different settings, there is no reliable data regarding RHD prevalence. Therefore, the aim of this systematic review and meta-analysis was to estimate the pooled prevalence of RHD in Ethiopia. PubMed/Medline, SCOPUS, HINARI, and Google Scholar databases were used to search for peer-reviewed articles. Articles published in English between the years 1992 and 2022 September were considered. The pooled prevalence of RHD was calculated using a random-effect model at a 95% confidence interval, including the weight of each study. Finally, statistical meta-analysis STATA version 16.0 software was used to calculate the pooled prevalence of RHD.A total of twelve cross-sectional studies were included in the meta-analysis. Individual study prevalence ranges from 0.32 to 32.78%. The pooled prevalence of RHD was 3.19% (95% CI: 1.46–5.56%). The prevalence was higher among the population who visited hospitals at 5.42% (95% CI: 1.09–12.7%) compared to schoolchildren at 0.73% (95% CI: 0.30–1.34%) and community-based studies at 3.83% (95% CI: 3.16–4.55%). Addis Ababa had the lowest prevalence of RHD (0.75% (95% CI: 0.38–1.25%), whereas the highest prevalence was observed in the Amhara region (8.95% (95% CI: 7.21–11.06%). A significant variation in the overall estimated prevalence of RHD was not observed between males and females.

**Trial registration** Protocol registration (PROSPERO): CRD42021251553, Date of registration May 28 2021

**Supplementary Information:**

The online version contains supplementary material available at 10.1186/s40794-023-00192-y.

## Introduction

Rheumatic Heart Disease (RHD) is the most common cause of group Astreptococcal-related cardiovascular morbidity, though it is virtually eliminated in developed nations [1]. Globally, around 33 million people are living with the disease, of which sub-Saharan Africa, South Asia, and Oceania account for the highest burden of the disease [2, 3]. In Africa, it is one of the most common causes of heart failure and carries a harsh prognosis due to the absence of specialized centers and the availability of cardiac surgery [4–7]. Different studies in Africa have reported that RHD accounts for up to 34% of hospital admissions related to cardiovascular disease and it is the most frequent cause of morbidity among children and young people [8]. The etiologic agent for RHD is Group A streptococcal bacterial infection, which causes acute rheumatic fever and later on advances to cause the heart muscle to swell and inflame through narrowing of the valves [9, 10].

Factors contributing to an increased prevalence of RHD in developing nations include household overcrowding, poor hygiene, and limited access to medical services [11, 12]. Strong RHD research and development initiatives have now led to notable modifications in the elimination of the disease in industrialized nations [4].

Even though global, regional, and national burdens of RHD are reported at different times, it remains difficult to estimate the prevalence of RHD in Ethiopia. The problem could be caused by the absence of a routine RHD screening plan in Ethiopian health policy, as well as the small number of studies done [10].

In Ethiopia, various studies have been conducted to estimate the prevalence of RHD in different contexts. A community-based study done in rural Ethiopia using trans-thoracic echocardiography indicated that 37.5% of the participants were found to have RHD [13]. Another multisite echocardiography-based study done among schoolchildren shows that 19 cases per 1000 children have evidence of the disease [14]. Like any other low-income country, Ethiopia is severely affected by RHD [15]. To the extent of our knowledge, except for a few fragmented studies, there has been no large population-based study or systematic review and meta-analysis done in this country in order to assess the pooled prevalence of RHD among all populations. Thus, the main aim of this systematic review and meta-analysis was to estimate the pooled prevalence of RHD in Ethiopia, and the findings might help policymakers revise policy.

## Material and methods

### Outcome variables

A data extraction tool was utilized to create data from each article chosen for review, which was then presented in a table in accordance with the PRISMA protocol. The primary outcome of this review was to estimate the quantitative pooled prevalence of RHD and the secondary outcome was to identify, if any, differences among community-based studies and institutional studies (school and hospital) and investigate any regional and gender variation regarding the prevalence of RHD in Ethiopia.

### Eligibility criteria

Studies reporting the prevalence of RHD that were conducted in Ethiopia and published in English between the periods of 1992 and 2022 September were considered. Age restrictions were not used during the article retrieval process. Studies that estimated the prevalence of RHD in screening programs at schools, hospitals, and communities were included. Studies that used both echocardiographic and auscultation as methods of screening modalities were considered. Cross-sectional studies with clear objectives and methods that have full text are included in the study. Degenerative heart valve disease, rheumatologic conditions other than RHD or solely focused on acute rheumatic fever, editorials, commentaries, and case reports were excluded from the study.

### Search strategy

The reviewed studies were accessed through electronic web-based search strategies from PubMed/Medline, SCOPUS, HINARI, and Google Scholar.

A three-step search strategy was utilized in this review. An initial limited search of PubMed, SCOPUS, and HINARI was undertaken, followed by analysis of the text words contained in the title and abstract, and of the index terms used to describe the article. A second search was undertaken across all included databases using all identified keywords and index terms. Finally, the reference list of all identified reports and articles was searched for additional studies, and only English-language studies were included.

The following search terms were used by using subject headings (MeSH in Pub Med/MEDLINE) for each database and free-text words for the concepts of "Prevalence," "Epidemiology","Cross-SectionalStudies","Rheumatic Heart Disease,""Child," "Adolescent,""Adult","Community-Based Participatory Research,""Hospital-Based,""Ethiopia,""Africa South of the Sahara” Boolean operators such as (AND, OR) were used to narrow the search. The reference lists of included studies and related reviews as indicated in Table 1 (Additional file 1).

The initial search was done by experienced team members with searching and screening of titles; abstracts and full texts were conducted independently by two reviewers (KH, SM). In the case of disagreements, the third author (AW) was invited and involved to reach a consensus.

### Risk of bias assessment

The risk of bias assessment tool adopted from Hoy et al. was used as a quality assessment checklist for critical appraisal of the selected articles [16]. The risk of bias assessment was done by two authors (SM and KH). The tool includes nine items, with a maximum score of nine and a minimum score of zero. The overall risk of bias has been ranked into three categories: low, moderate, and high risk. A summary of the risk of bias for all the twelve included articles with a justification of the rating for each item is provided in Table 2 (Additional file 2).

### Data extraction and outcome of interest

Duplicates were removed; full-text studies were retrieved for the selected abstracts; and eligible studies that lacked full text (only abstracts) from the primary authors were contacted through their email address. Individual studies yielded the following information:

1. Author name and years of publication; study design; age of the participants.
2. Diagnostic modalities.
3. Prevalence estimate reported by community, school, hospital, sex, and region.

### Reliability of data

#### Analysis of the Data

The primary outcome of this review was the pooled prevalence of RHD in Ethiopia. The meta-analysis was done by using STATA version 16.0 and a random effects model, weighted by the inverse of variance. Heterogeneity between estimates was assessed using the I^2^ statistic (percentage of residual variation attributed to heterogeneity); an I^2^ value above 75% was taken as considerable heterogeneity within the studies [17].

The pooled prevalence of RHD and its 95% confidence intervals (CIs) were reported in the pooled analysis. Subgroup analysis was done based on study settings (community, school, and hospital), gender, and regions of the country. The trend of RHD prevalence with publication years was and sensitivity analysis for possible sources of heterogeneity and publication bias was done by visual assessment of the funnel plot, and small-study effect was assessed by Egger’s test.

## Result

### Study selection

**Fig. 1:**
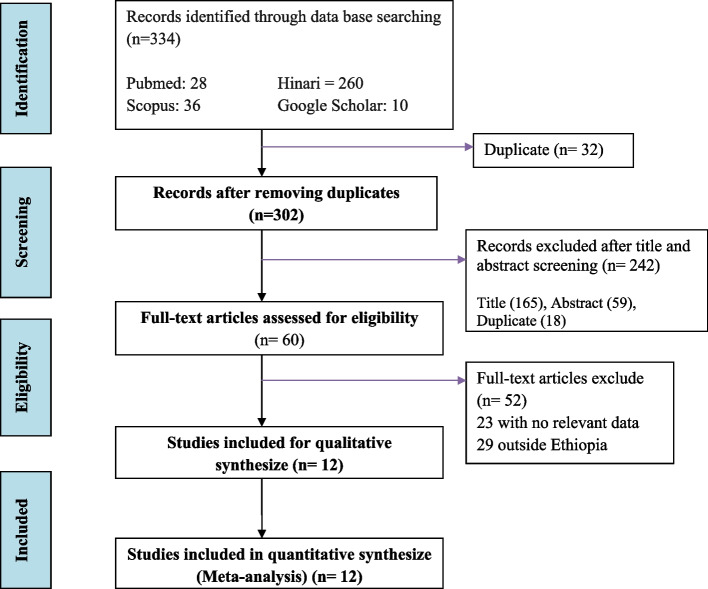
PRISMA flow chart for identifications of studies included in systematic review and meta-analysis on prevalence of rheumatic heart diseases in Ethiopia

Overall, 334 studies were initially retrieved from different databases. After removing the duplicates, 302 studies were screened and a total of 60 eligible abstracts were screened by inclusion criteria, followed by screening of full-text studies. Finally, 12 studies satisfying the eligibility criteria were included in the meta-analysis of rheumatic heart disease (Fig. 1).

### Characteristics of studies included in the Meta-Analysis

Twelve studies with a total sample size of 33,009 participants have been included in the meta-analysis. The prevalence of RHD is nearly similar among sexes, with 1.89 (95% CI: 0.63–3.16) and 1.95 (95% CI: 0.54–3.35) for males and females, respectively. The majority of the studies (41.6%) were conducted in Oromia, followed by Addis Ababa (25%), while the remaining studies were conducted in South Nation Nationality and People (SNNP) (16.6%), Amhara (8.24), and national-level (8.4%).Two studies by Bezaye & Henok [18] and Gemechu et al. [19] included pediatric ages between 2 months and 14 years, while the studies conducted by Bacha et al. and Moges et al. included pregnant populations [20, 21].

Regarding study setting, six studies were hospital-based [18–23]; the other four studies were school-based [14, 24, 25, 26]; and two studies [13, 27] were community-based studies (Table 1). To diagnose RHD, WHO echocardiography criteria for case definition, the American Society of Echocardiography with the European Association of Echocardiography standard and the World Heart Federation (WHF) criteria were used. All of the studies used a cross-sectional study design, and eleven studies used echocardiography, while one study used auscultation [24] as a method of diagnostic approach.

**Table 1:** Characteristics of the individual studies included in the systematic review and meta-analysis on prevalence of rheumatic heart diseases in Ethiopia

Authors	Publicati on Year	Study design	Region_ Ethiopia	Study Setting	Age of Participant (in Years)	Sample size	No. of RHD	Prevalence (%)	Diagnostic Method
Zelalem et al. [23]	2022	Cross Sectional	Amhara	Hospital Based	6 -103	849	76	18.2	Echocardiography
Bacha et al. [20]	2019	Cross Sectional	AdissAbaba	Hospital Based	18 -40	398	9	2.3	Echocardiography
Moges et al. [21]	2021	Cross Sectional	Oromia	Hospital Based	mean age was 29 years	4584	29	0.6	Echocardiography
Engel et al. [27]	2015	Cross Sectional	Oromia	Community based	4—24	2000	61	3.05	Echocardiography
Gemechu et al. [13]	2017	Cross Sectional	Oromia	Community based	6—25	987	56	5.7	Echocardiography
Mulatu et al. [26]	2015	Cross Sectional	Oromia	School based	5 -15	1874	6	0.32	Echocardiography
Oli et al. [24]	1992	Cross Sectional	SNNP	School Based	5–19	3235	15	0.46	Auscultation
Yadeta et al. [14]	2016	Cross Sectional	National	School based	6–18	3238	59	1.8	Echocardiography
Habte et al. [22]	2010	Cross Sectional	Oromia	Hospital based	Adult (mean age was 31.4 years)	781	256	32.8	Echocardiography
Bezaye&Henok [18]	2019	Cross Sectional	SNNP	Hospital Based	2 months—14 years	2000	75	3.75	Echocardiography
Gebremariam&Moges [19]	2016	Cross Sectional	Addis Ababa	Hospital Based	2 months—14 years	3672	19	0.52	Echocardiography
Oli&Porteous [25]	1999	Cross Sectional	Addis Ababa	School Based	school children	9388	60	0.64	Echocardiography

### Risk of bias assessment

#### Assessment of publication Bias

**Fig. 2:**
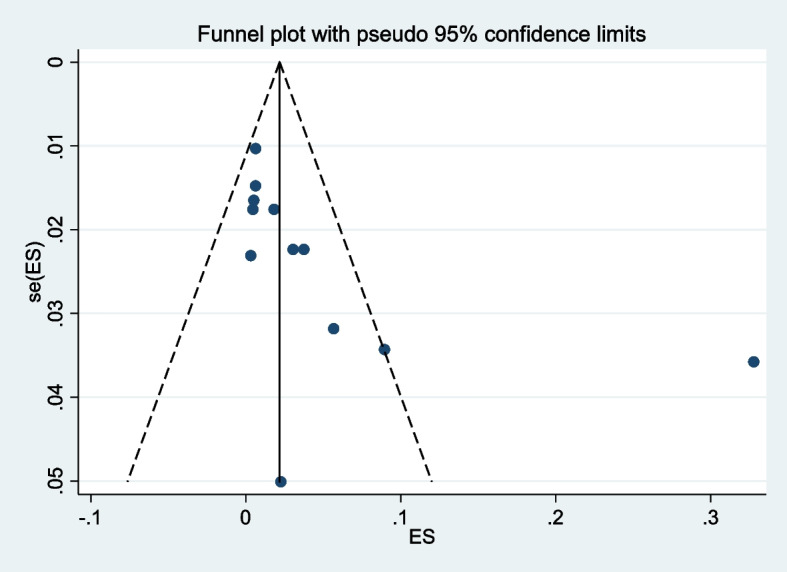
Funnel plot for assessment of publication bias across studies

Publication bias was evaluated using Egger’s test. The publication bias funnel plot shows that there is an apparently symmetrical distribution (Fig. 2), and Egger's regression test was found to be significant with a *p*-value of *P* = 0.001. Thus, the test provides evidence for no or undetected publication bias in the included studies. The estimated bias coefficient was 9.939 (Egger bias B = 9.939 (95% CI: 5.548–14.330; *p* = 0.001)) with a standard error of 1.971, as indicated in Table 2.

**Table 2:** Assessment of publication bias using egger’s test among studies

Number of studies = 12			Root MSE = 4.157			
Std_Eff	Coef	Std.Err	T	*P*>t	[95%Conf	Interval]
Slope	-0.005	0.003	-1.680	0.124	-0.012	0.002
Bias	9.939	1.971	5.040	0.001	5.548	14.330

Test of H0: no small-study effects *P* = 0.001

Note: Data input format theta se_theta assumed, Egger's test for small-study effects: Regress standard normal deviate of intervention effect estimate against its standard error

#### Prevalence of rheumatic heart disease

The estimated pooled prevalence of RHD, reported by twelve primary studies using a fixed effect model, showed significant heterogeneity between the studies.

**Fig. 3:**
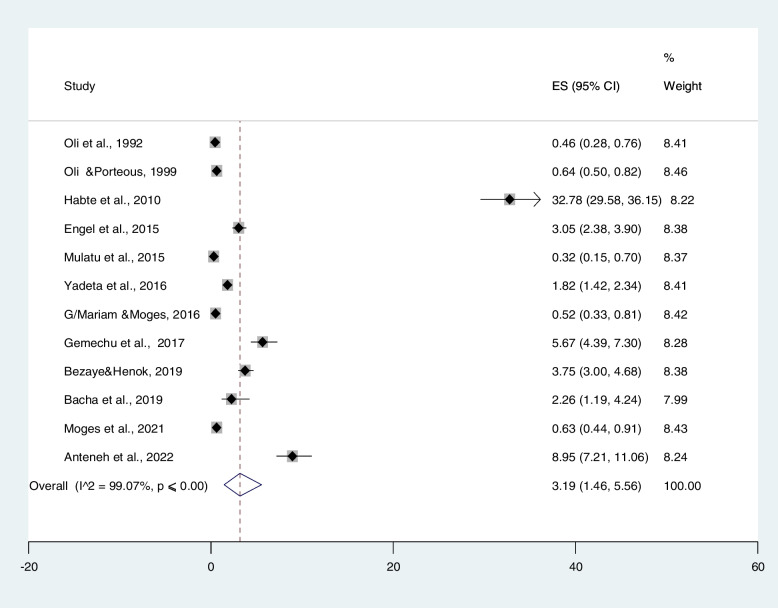
Forest plot on studies that quantitatively assessed pooled prevalence of rheumatic heart disease in Ethiopia

Accordingly, a random-effect model was used to estimate pooled prevalence. In primary studies (*n* = 12), the prevalence of RHD ranged from 0.32% in a study conducted by Mulatu et al. [26] to 32.78% in a study conducted by Habte et al. [22]. In the random-effect model, the prevalence of RHD was 3.19% (95% CI: 1.46–5.56%) with significant heterogeneity between the studies (I2 = 99.07%), *p* ≤ 0.001) (Fig. 3).

### Subgroup analysis and investigation of heterogeneity

#### Prevalence of RHD based on study setting

**Fig. 4:**
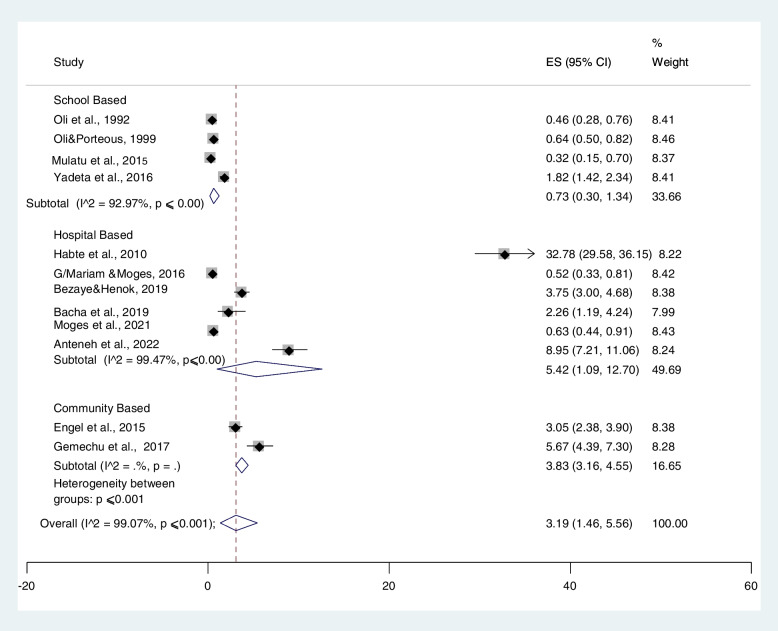
Subgroup analysis on the prevalence of rheumatic heart disease among different study settings

Based on study location, places of study were categorized into three settings: school, hospital, and community-based. The highest prevalence was observed in the population who visited hospitals, 5.42% (95% CI: 1.09%–12.70%) compared with schoolchildren (0.73% (95% CI: 0.30–1.34%) and community-based studies at 3.83% (95% CI: 3.16–4.55). (Fig. 4). Subgroup analysis was performed per study setting, and potential heterogeneity was detected in the prevalence estimates of RHD across studies (I^2^ range: 99.47–99.97%; all *p* ≤ 0.001).

#### Prevalence of RHD based on region

**Fig. 5:**
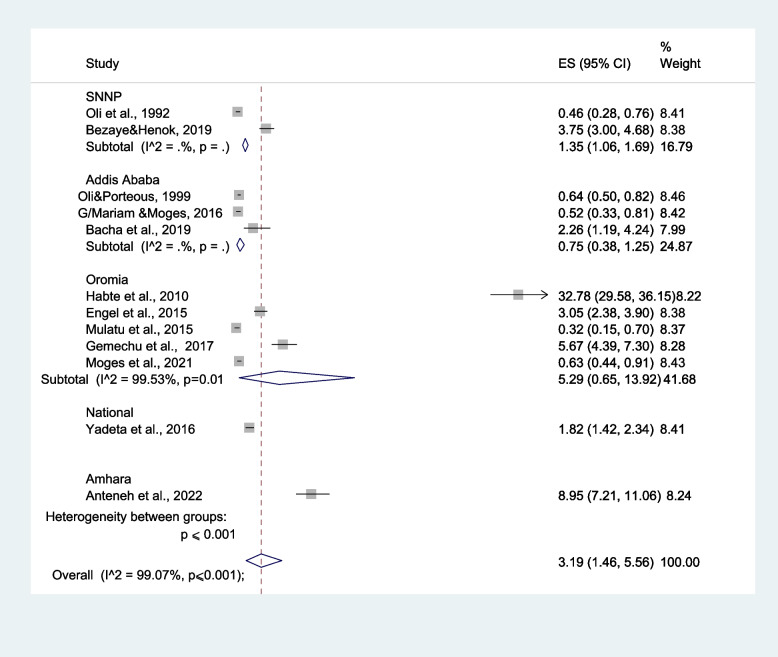
Subgroup analysis on the prevalence of rheumatic heart disease among different regions of Ethiopia

Based on the administrative division of Ethiopia, the study regions were grouped into five regions: SNNP, Amhara, Addis Ababa, Oromia, and National (studies done in two or more regions). RHD was less prevalent in Adiss Ababa (0.75% (95% CI: 0.38%–1.25%) compared with Amhara (8.95% (95% CI: 7.21–11.06%) (Fig. 5). In the region-based subgroup analysis, potential heterogeneity was detected in the prevalence estimates of RHD across studies (I^2^ range: 99.07–99.53%; all *p* ≤ 0.001).

#### Prevalence of RHD based on gender

**Fig. 6:**
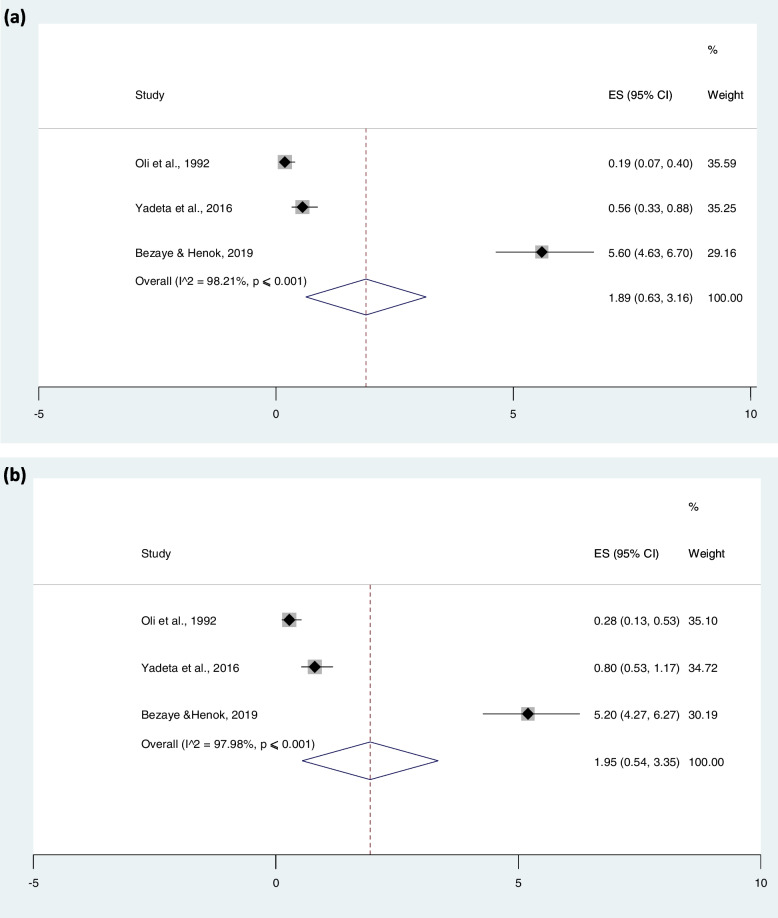
Prevalence of rheumatic heart disease among males (**a**) and females (**b**) in Ethiopia

Subgroup studies based on gender were done for three studies, with a total population of 8476; the pooled prevalence of RHD among males was 1.89 (95% CI: 0.63–3.16%) and females was 1.95 (0.54–3.35), which was almost similar as indicated in Fig. 6.

#### Trends of RHD prevalence through year of publication

**Fig. 7:**
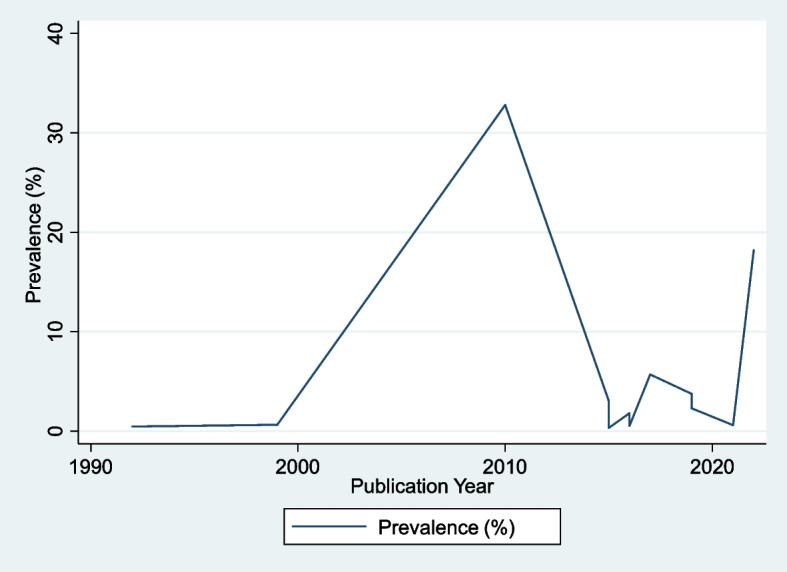
Trends of rheumatic heart disease prevalence by year of publication in Ethiopia

The prevalence of RHD did not change between 1990 and 2000 but increased significantly from 0.64% to 32.78% between 2000 and 2010, and this pattern continued after 2020 (Fig. 7).

## Discussion

In the current systematic review and meta-analysis, the reviewers have explored published papers from different sources and finally included 12 primary studies that involved a total of 33,009 study participants. During this process, reviewers have identified a pooled prevalence of RHD as being 3.19% among the all-sex and age-inclusive Ethiopian population. For screening purposes, both the auscultation and echocardiography methods of diagnosis are employed in the study. Even though echocardiography is the gold standard for screening RHD with the best imaging contrast, careful clinical auscultation using a stethoscope remains a valuable tool for cardiac diagnosis in low-income countries [28, 29].

In terms of RHD prevalence patterns by year of publication, the prevalence of RHD did not change between 1990 and 2000 but climbed drastically between 2000 and 2010, from 0.64% to 32.78%, and this trend has continued until 2020. This could be due to the fact that the Ethiopian government is currently working to improve Ethiopians' health and well-being by providing a comprehensive package of high-quality preventive, curative, and rehabilitative non-communicable disease services in an equitable manner [30].

The identified pooled prevalence of the current systematic review and meta-analysis was compared with different studies conducted at different times by different researchers. Accordingly, the current 3.19% pooled prevalence in the Ethiopian context is found to be lower as compared to the pooled prevalence reported by the study conducted in endemic areas by Rothenbuhler et al. and the global systemic review done by Jean Jacques et al., where the prevalence was 12.9% and 26.1%, respectively [31, 32]. In contrast, the current finding was slightly greater by a percentage than the South Asian study by Lamichhane P et al. in Nepal [33], which was 2.79 percent of a comparable population. This could be due to the Nepalese government's dedication and the community's involvement through the development of an RHD registry, comprehensive program evaluation, and improving the quality and safety of Benzathine Penicillin supplies [34].

In the sub-group meta-analysis, the prevalence of RHD among individuals who visited hospitals was found to be 5.42%, which is more than 7 times higher compared to the school-based study. The possible reason for the heterogeneity among hospital-based studies and school- or community-based studies could be the straight-forward difference among study participants. The participants in hospital settings could be diseased individuals who sought healthcare services, whereas the school-level participants are notably healthy individuals. Moreover, the researchers have deployed a document review for data collection at hospitals and, on the other hand, have used face-to-face interviews for both school and community-based studies. Therefore, differences in the method of data collection could also contribute to the variation in recorded prevalence.

In a separate view, the school-based pooled prevalence of RHD according to this study was 0.73%, indicating a reasonable difference from the hospital or community-based pooled prevalence. In any case, the school-based pooled prevalence was found to be comparably similar to the pooled prevalence of RHD that was reported in Nepal among schoolchildren [35]. On the contrary, the current school-based pooled RHD prevalence was higher when compared with the pooled prevalence of 0.04% in the same case among Iranian schoolchildren [36]. Even though the reasons for the disparity in the prevalence among the studies have not been clearly established, literatures ambiguously forwards the possible reasons to be differences in shortage of basic needs, overcrowding, malnutrition, low level of awareness about the disease, and accessibility of healthcare services for screening, early identification, and treatment of the disease in the Ethiopian context [37].

In terms of geographical distribution, Amhara had the highest prevalence of RHD, with Oromia and SNNP ranking in second and third places, respectively. The possible explanations for the variation include differences in population density between regions, as well as levels of health service utilization and related water supply that is particularly low in the Amhara region compared to Oromia and SNNP regions. However, as the heterogeneity in the areas has been identified to be higher, the prevalence might not represent a real higher prevalence. Yet, the variability in the characteristics of the study setting, such as dense population, existence of many disadvantaged populations, and relatively lower healthcare service coverage in Oromia and Ahmara regions, could support the result of the current review.

Additionally, the study done by Bezaye and Henok [18] included pediatric ages between 2 months and 14 years, while the studies conducted by Bacha et al. and Moges et al. [20, 21] included pregnant populations, which would grossly contribute to the variation among the studies.

Finally, the current review has revealed the overall sex distribution of RHD among males and females to be the same. On the contrary to this, previous studies have suggested the likelihood of a higher prevalence of RHD in females than in males of the same age for several reasons. To that end, previous research has identified the poor health-seeking behaviors of females and women who are burdened with home activities that expose them to indoor smokes produced during cooking. The higher exposure further increases the risk of upper respiratory infections among women [38]. Nowadays, however, the aforementioned risk factors are relatively equally distributed among populations independent of sex due to equitable healthcare, modern cooking technologies and indoor or community-based health services in the current study setting, unlike the report from Nepal, where the prevalence of RHD is higher among females [39].

## Strength and limitations

Data extraction and critical appraisal were done by two experienced reviewers. Besides this, sub-group analysis and sensitivity analysis were implemented to assess the degree of heterogeneity. However, the current studies lack detailed descriptions of the classification of cases as definite RHD or borderline RHD based on echocardiographic findings.

## Conclusion

This review revealed that the prevalence of rheumatic heart disease is high in Ethiopia. The highest prevalence was observed among clients who visited hospitals compared to individuals screened at the school and community level. In terms of geographical distribution, Amhara had the highest prevalence of RHD, followed by Oromia and SNNP, respectively. There is no significant sex difference in acquiring the disease. The review has also implicated that the study related to RHD prevalence has not been covered in all regions of Ethiopia. Therefore, the authors call for further studies to cover the overall geography of the country and further observational studies to explicitly characterize RHD and support policymakers.

### Supplementary Information

**Additional file 1: Table 1.** Searching strategy and information sources.

**Additional file 2:** **Table 2.** Critical AppraisalTool.

## Data Availability

The datasets supporting the conclusions of this article are included in the article.

## Abbreviations

CI: Confidence interval
ES: Effect size
OR: Odds ratio
RHD: Rheumatic Heart Disease
SNNP: Southern Nation Nationality and People
WFH: World Heart Federation
WHO: World Health Organization
