# Supplementary material for: The prevalence of rheumatic heart disease in Ethiopia: a systematic review and meta-analysis
Source: Trop Dis Travel Med Vaccines. 2023 Oct 13;9:16. doi: 10.1186/s40794-023-00192-y (PMC10571304; doi:10.1186/s40794-023-00192-y)
Supplement: Supplementary file 1 — Additional file 1: Table 1. Searching strategy and information sources. [file 40794_2023_192_MOESM1_ESM.docx]

Table 1 Searching strategy and information sources

| **Databases** | **MeSH heading** | **Searching combination** | **Searching Date** | **Results** |
| --- | --- | --- | --- | --- |
| PubMed/Medline | Rheumatic Heart Disease, Prevalence, Cross-Sectional Studies, Epidemiology, Ethiopia | ("rheumatic heart disease"[All Fields] OR (("rheumatoid"[All Fields] OR "rheumatoids"[All Fields]) AND ("heart"[MeSH Terms] OR "heart"[All Fields] OR "hearts"[All Fields] OR "heart s"[All Fields]) AND ("fever"[MeSH Terms] OR "fever"[All Fields] OR "fevers"[All Fields]))) AND ("prevalence"[All Fields] OR "epidemiology"[All Fields]) AND "ethiopia"[MeSH Terms] | September 30, 2022 | 28 |
| Scopus | Prevalence, Rheumatic Heart Disease, Community-based, Institution based, Ethiopia | ( TITLE-ABS-KEY ( prevalence ) AND TITLE-ABS-KEY ( "Rheumatic Heart Disease" ) AND TITLE-ABS-KEY ( community-based ) OR TITLE-ABS-KEY ( institution-based ) OR TITLE-ABS-KEY ( ethiopia ) ) | September 30, 2022 | 36 |
| Hinari | Prevalence, Cross sectional Studies, Epidemiology, rheumatic heart disease, Ethiopia | ((TitleCombined:(Prevalence)) OR (Cross Sectional Studies) OR (Epidemiology)) AND (rheumatic heart disease) AND ("ethiopia") | September 30, 2022 | 260 |
| **Others** | | | | |
| Google Scholar |  | allintitle: prevalence rheumatic heart disease Ethiopia |  | 10 |
