# Supplementary material for: The prevalence of rheumatic heart disease in Ethiopia: a systematic review and meta-analysis
Source: Trop Dis Travel Med Vaccines. 2023 Oct 13;9:16. doi: 10.1186/s40794-023-00192-y (PMC10571304; doi:10.1186/s40794-023-00192-y)
Supplement: Supplementary file 2 — Additional file 2: Table 2. Critical AppraisalTool. [file 40794_2023_192_MOESM2_ESM.docx]

## Table 2: Critical Appraisal Tool

Risk of bias assessment

Risk of bias is done using the Hoy et al prevalence study quality assessment tool which ,the tool has major three components and graded as low ,moderate and high risk.

(1) Low risk (0-3), (2) moderate risk (4-6) finally high risk (7-9) Adapted from Hoy et al (2012)

| **First Author (year)** | **ENTRY** | **JUDGEMENT** | **DESCRIPTION** |
| --- | --- | --- | --- |
| **Anteneh (2022)** | ***External Validity*** |  |  |
| **1** | Was the study’s target population **a close representation** of the national population in relation to relevant variables, e.g. age, sex, occupation? | 1 | The study particpant age was greter than 24 |
| **2** | Was the sampling frame a **true or close representation** of the target population? | 0 | The study samples were drawn from the target population |
| **3** | Was some form of **random selection** used to select the sample, OR, was a census undertaken? | 0 | All patients are included after echocardiography diagnostic  tests |
| **4** | Was the likelihood of **non-response biasminimal?** | 0 | All 849 patients include cardiac clinic are included |
|  | ***Internal Validity*** |  |  |
| **5** | Were data collected **directly from the subjects** (as opposed to a proxy)? | 1 | Data was collected directly from the patients |
| **6** | Was an acceptable case definition used in the study? | 0 | There is clear case defination |
| **7** | Was the study instrument that measured the parameter of interest shown to have **reliability and validity (if necessary)**? | **1** | Not well mentioned |
| **8** | Was the **same mode of data collection** used for all subjects? | **0** | Similar modeof datacollection were used |
| **9** | Were the **numerator(s) and denominator(s)** for the parameter of interest appropriate? | **0** | Yes,approperate parameters used |
| **Overall risk of Bias** | | **3- low risk** |  |
| **First Author (year)** | **ENTRY** | **JUDGEMENT** | **DESCRIPTION** |
| **Engel (2015)** | External Validity |  |  |
| **1** | Was the study’s target population a close representation of the national population in relation to relevant variables, e.g. age, sex, occupation? | 1 | 0nly Individuals from 4 - 24 years of age were included in the study. |
| **2** | Was the sampling frame a true or close representation of the target population? | 1 | The sampling frame was a list of one particular area within the overall target population |
| **3** | Was some form of random selection used to select the sample, OR, was a census undertaken? | 0 | Appropriate randomized selection was taken |
| **4** | Was the likelihood of non-response bias minimal? | 0 | The response rate for the study was 99% |
|  | Internal Validity |  |  |
| **5** | Were data collected directly from the subjects (as opposed to a proxy)? | 0 | Data was collected directly from pupils from school |
| **6** | Was an acceptable case definition used in the study? | 0 | Clear case definition was used |
| **7** | Was the study instrument that measured the parameter of interest (e.g. prevalence of low back pain) shown to have reliability and validity (if necessary)? | 0 | The researcher used evidence-based echocardiographic diagnostic criteria of the World Heart Federation (WHF). |
| **8** | Was the same mode of data collection used for all subjects? | 0 | Use similar mode of data collection techniques |
| **9** | Were the numerator(s) and denominator(s) for the parameter of interest appropriate? | 0 | Both numerator and denominator were used appropriatly |
|  | Overall risk of Bias | **2 (low risk)** |  |

| **First Author (year)** | **ENTRY** | **JUDGEMENT** | **DESCRIPTION** |
| --- | --- | --- | --- |
| **Mulatu (2016)** | ***External Validity*** |  |  |
| **1** | Was the study’s target population **a close representation** of the national population in relation to relevant variables, e.g. age, sex, occupation? | **1** | The study take place in Shashemene among Primary School Students , unable to represent the whole Ethiopia population |
| **2** | Was the sampling frame a **true or close representation** of the target population? | **1** | The sampling frame was a list of one particular area within the overall target population |
| **3** | Was some form of **random selection** used to select the sample, OR, was a census undertaken? | **0** | The study uses appropriate sampling methods |
| **4** | Was the likelihood of **non-response biasminimal?** | **1** | response rate was not reported |
|  | ***Internal Validity*** |  |  |
| **5** | Were data collected **directly from the subjects** (as opposed to a proxy)? | **0** | data collected **directly from the subjects** |
| **6** | Was an acceptable case definition used in the study? | **0** | Yes, acceptable case definition were used in the document |
| **7** | Was the study instrument that measured the parameter of interest (e.g. prevalence of low back pain) shown to have **reliability and validity (if necessary)**? | **0** | The 2006 WHO echocardiography criteria  for case definition of RHD were used. |
| **8** | Was the **same mode of data collection** used for all subjects? | **0** | Across all individuals the same mode of data collection were used |
| **9** | Were the **numerator(s) and denominator(s)** for the parameter of interest appropriate? | **0** | Yes, appropriate parameters were used |
|  | Overall risk of Bias | **3- low risk** |  |
| **First Author (year)** | ENTRY | **JUDGEMENT** | **DESCRIPTION** |
| **Habte (2010)** | External Validity |  |  |
| **1** | Was the study’s target population a close representation of the national population in relation to relevant variables, e.g. age, sex, occupation? | **0** | The study include age groups ,sex and place of recidence |
| **2** | Was the sampling frame a true or close representation of the target population? | **0** | **Yes** |
| **3** | Was some form of random selection used to select the sample, OR, was a census undertaken? | **0** | The study use the whole study popollation (censes take place) |
| **4** | Was the likelihood of non-response bias minimal? | **1** | records of 56 patients were incomplete |
|  | Internal Validity |  |  |
| **5** | Were data collected directly from the subjects (as opposed to a proxy)? | **1** | Data were extracted from the patient charts. |
| **6** | Was an acceptable case definition used in the study? | **1** | Cases were not well defined |
| **7** | Was the study instrument that measured the parameter of interest (e.g. prevalence of low back pain) shown to have reliability and validity (if necessary)? | **0** | Pretest was done |
| **8** | Was the same mode of data collection used for all subjects? | **1** | Data were collected only from patient chart |
| **9** | Were the numerator(s) and denominator(s) for the parameter of interest appropriate? | **0** | Yes, appropriate parameters were used |
|  | Overall risk of Bias | **4 moderate risk** | More than 3 domians were at high risk |

| **First Author (year)** | **ENTRY** | **JUDGEMENT** | **DESCRIPTION** |
| --- | --- | --- | --- |
| **Moges (2021)** | **External Validity** |  |  |
| **1** | **Was the study’s target population a close representation of the national population in relation to relevant variables, e.g. age, sex, occupation?** | **1** | **The study do not represent the whole popolation it represent only pregnant mothers on ANC follow up** |
| **2** | **Was the sampling frame a true or close representation of the target population?** | **1** | **The sampling frame was a list of only pregnant women with**  **VHD managed at JUMC during the study period** |
| **3** | **Was some form of random selection used to select the sample, OR, was a census undertaken?** | **1** | **The study particpants taken purposively** |
| **4** | **Was the likelihood of non-response biasminimal?** | **1** | **response rate was not reported** |
|  | **Internal Validity** |  |  |
| **5** | **Were data collected directly from the subjects (as opposed to a proxy)?** | **0** | **data collected directly from the subjects** |
| **6** | **Was an acceptable case definition used in the study?** | **1** | **Cases were not well defined** |
| **7** | **Was the study instrument that measured the parameter of interest (e.g. prevalence of low back pain) shown to have reliability and validity (if necessary)?** | **0** | **Two-dimensional trans-thoracic**  **Echocardiography was used to diagnose cardiac**  **disease and was performed according to American**  **Society of Echocardiography Guideline** |
| **8** | **Was the same mode of data collection used for all subjects?** | **0** | **Across all individuals the same mode of data collection were used** |
| **9** | **Were the numerator(s) and denominator(s) for the parameter of interest appropriate?** | **0** | **Yes, appropriate parameters were used** |
|  | **Overall risk of Bias** | **5- moderate risk** | More than 3 domians were at high risk |
| **First Author (year)** | **ENTRY** | **JUDGEMENT** | **DESCRIPTION** |
| **Bacha (2019)** | **External Validity** |  |  |
| **1** | **Was the study’s target population a close representation of the national population in relation to relevant variables, e.g. age, sex, occupation?** | **1** | **The study include only pregnant popolation** |
| **2** | **Was the sampling frame a true or close representation of the target population?** | **0** | **Yes** |
| **3** | **Was some form of random selection used to select the sample, OR, was a census undertaken?** | **0** | **Systematic**  **random sampling was used to select the samples** |
| **4** | **Was the likelihood of non-response bias minimal?** | **0** | **5% of non-response rate was considered in the study** |
|  | **Internal Validity** |  |  |
| **5** | **Were data collected directly from the subjects (as opposed to a proxy)?** | **0** | **Yes ,data collected directly** |
| **6** | **Was an acceptable case definition used in the study?** | **1** | **Cases were not well defined** |
| **7** | **Was the study instrument that measured the parameter of interest (e.g. prevalence of low back pain) shown to have reliability and validity (if necessary)?** | **1** | **Not mentioned** |
| **8** | **Was the same mode of data collection used for all subjects?** | **0** | **yes** |
| **9** | **Were the numerator(s) and denominator(s) for the parameter of interest appropriate?** | **0** | **Yes, appropriate parameters were used** |
|  | **Overall risk of Bias** | **3 low risk** | **More than 3 domians were at high risk** |
| **First Author (year)** | **ENTRY** | **JUDGEMENT** | **DESCRIPTION** |
| **Gemechu (2016)** | ***External Validity*** |  |  |
| **1** | Was the study’s target population **a close representation** of the national population in relation to relevant variables, e.g. age, sex, occupation? | **1** | Only 6-25 years of age included |
| **2** | Was the sampling frame a **true or close representation** of the target population? | **1** | Jimma zone only cannot represent rural Ethiopia |
| **3** | Was some form of **random selection** used to select the sample, OR, was a census undertaken? | **1** | Initially purposive then systematic and no census |
| **4** | Was the likelihood of **non-response biasminimal?** | **0** | The response rate is 82% |
|  | ***Internal Validity*** |  |  |
| **5** | Were data collected **directly from the subjects** (as opposed to a proxy)? | **0** | Yes ,data collected directly |
| **6** | Was an acceptable case definition used in the study? | **0** | RHD on the basis of the WHF criteria |
| **7** | Was the study instrument that measured the parameter of interest (e.g. prevalence of low back pain) shown to have **reliability and validity (if necessary)**? | **1** | pretested was not done |
| **8** | Was the **same mode of data collection** used for all subjects? | **0** | Yes, subjects assesesd using a face-to-face interview |
| **9** | Were the **numerator(s) and denominator(s)** for the parameter of interest appropriate? | **0** |  |
|  | Overall risk of Bias | **4- moderate risk** |  |
| **First Author (year)** | ENTRY | **JUDGEMENT** | **DESCRIPTION** |
| **Bezaye (2019)** | External Validity |  |  |
| **1** | Was the study’s target population a close representation of the national population in relation to relevant variables, e.g. age, sex, occupation? | **1** | only children aged 2 months to 14 years were included |
| **2** | Was the sampling frame a true or close representation of the target population? | **0** | It accounts all pediatric individuals recorded in the hospital |
| **3** | Was some form of random selection used to select the sample, OR, was a census undertaken? | **0** | Censu was done |
| **4** | Was the likelihood of non-response bias minimal? | **0** | It was secondary data and complete census |
|  | Internal Validity |  |  |
| **5** | Were data collected directly from the subjects (as opposed to a proxy)? | **1** | It is from secondary data |
| **6** | Was an acceptable case definition used in the study? | **0** | All cause are well defined |
| **7** | Was the study instrument that measured the parameter of interest (e.g. prevalence of low back pain) shown to have reliability and validity (if necessary)? | **0** | New York Heart Association (NYHA)classification were used |
| **8** | Was the same mode of data collection used for all subjects? | **0** | data were collected using structured record review checklist |
| **9** | Were the numerator(s) and denominator(s) for the parameter of interest appropriate? | **0** |  |
|  | Overall risk of Bias | **2-low risk** |  |

| **First Author (year)** | **ENTRY** | **JUDGEMENT** | **DESCRIPTION** |
| --- | --- | --- | --- |
| **Oli K (1999)** | ***External Validity*** |  |  |
| **1** | Was the study’s target population **a close representation** of the national population in relation to relevant variables, e.g. age, sex, occupation? | **1** | Only Grade seven and eight students are included in the study. |
| **2** | Was the sampling frame a **true or close representation** of the target population? | **0** | The sampling from was a list of all populations in that target population |
| **3** | Was some form of **random selection** used to select the sample, OR, was a census undertaken? | **0** | Random selection is used to select both Government and private school |
| **4** | Was the likelihood of **non-response biasminimal?** | **0** | The response rate was 93% |
|  | ***Internal Validity*** |  |  |
| **5** | Were data collected **directly from the subjects** (as opposed to a proxy)? | **0** | Data was collected directly from students |
| **6** | Was an acceptable case definition used in the study? | **0** | There was clear case defination |
| **7** | Was the study instrument that measured the parameter of interest (e.g. prevalence of low back pain) shown to have **reliability and validity (if necessary)**? | **0** | The questionare were tested to assess the prevalence of RHD among Addis Ababa School children . |
| **8** | Was the **same mode of data collection** used for all subjects? | **0** | Information was collected through questioner that asess difrent parmeters like socio economic status |
| **9** | Were the **numerator(s) and denominator(s)** for the parameter of interest appropriate? | **0** | Appropriate numerator and denominator were used to calculate prevalence |
|  | Overall risk of Bias | **1 (low risk)** |  |
| **First Author (year)** | ENTRY | **JUDGEMENT** | **DESCRIPTION** |
| **Yadeta (2016)** | External Validity |  |  |
| **1** | Was the study’s target population a close representation of the national population in relation to relevant variables, e.g. age, sex, occupation? | **0** | Includes all regions of Ethiopia with relevant variables |
| **2** | Was the sampling frame a true or close representation of the target population? | **0** | a list of almost every individual within the target population are included in the sampling frame |
| **3** | Was some form of random selection used to select the sample, OR, was a census undertaken? | **0** | The study try to sample every individual with in the target population |
| **4** | Was the likelihood of non-response bias minimal? | **0** | The response rate was 98.1% |
|  | Internal Validity |  |  |
| **5** | Were data collected directly from the subjects (as opposed to a proxy)? | **0** | Data were collected directly from the study subjects |
| **6** | Was an acceptable case definition used in the study? | **0** | Used standard case definitions |
| **7** | Was the study instrument that measured the parameter of interest (e.g. prevalence of low back pain) shown to have reliability and validity (if necessary)? | **1** | Instrument was not validated or reliability test were not done |
| **8** | Was the same mode of data collection used for all subjects? | **0** | same mode of data collection used for all subjects |
| **9** | Were the numerator(s) and denominator(s) for the parameter of interest appropriate? | **0** | Appropriate numerator and denominator was used to calculate prevalence |
|  | Overall risk of Bias | **1 (low risk)** |  |

| **First Author (year)** | **ENTRY** | **JUDGEMENT** | **DESCRIPTION** |
| --- | --- | --- | --- |
| **Oli K (1992)** | ***External Validity*** |  |  |
| **1** | Was the study’s target population **a close representation** of the national population in relation to relevant variables, e.g. age, sex, occupation? | **0** | All age group and both sex founed in Butajira elementery school were included |
| **2** | Was the sampling frame a **true or close representation** of the target population? | **1** | Not clear |
| **3** | Was some form of **random selection** used to select the sample, OR, was a census undertaken? | **0** | Census was under take |
| **4** | Was the likelihood of **non-response biasminimal?** | **0** | The response rate was 92.8% |
|  | ***Internal Validity*** |  |  |
| **5** | Were data collected **directly from the subjects** (as opposed to a proxy)? | **0** | The data was taken from school children |
| **6** | Was an acceptable case definition used in the study? | **1** | There is no clear statement of case defination for RHD |
| **7** | Was the study instrument that measured the parameter of interest (e.g. prevalence of low back pain) shown to have **reliability and validity (if necessary)**? | **1** | There was no clear report on reliability and validity of the nstrument |
| **8** | Was the **same mode of data collection** used for all subjects? | **1** | Initial screening was done with a high index of suspicion. |
| **9** | Were the **numerator(s) and denominator(s)** for the parameter of interest appropriate? | **0** | Appropriate numrator and denominator were used |
|  | Overall risk of Bias | **4( moderate risk)** |  |

| **First Author (year)** | **ENTRY** | **JUDGEMENT** | **DESCRIPTION** |
| --- | --- | --- | --- |
| **Gebremariam (2016)** | ***External Validity*** |  |  |
| **1** | Was the study’s target population **a close representation** of the national population in relation to relevant variables, e.g. age, sex, occupation? | **0** | It adress pediatrics age group who come from diffrent corner of Ethiopia . |
| **2** | Was the sampling frame a **true or close representation** of the target population? | **1** | Not clearly indicated |
| **3** | Was some form of **random selection** used to select the sample, OR, was a census undertaken? | **1** | The study samples were purposivelly selected |
| **4** | Was the likelihood of **non-response biasminimal?** | **1** | Unclear |
|  | ***Internal Validity*** |  |  |
| **5** | Were data collected **directly from the subjects** (as opposed to a proxy)? | **1** | Discharge recordes was taken |
| **6** | Was an acceptable case definition used in the study? | **0** | There was clear case defination |
| **7** | Was the study instrument that measured the parameter of interest shown to have **reliability and validity (if necessary)**? | **0** | Data were extracted from the patient charts and were filled into the questionnaire format by the investigators. |
| **8** | Was the **same mode of data collection** used for all subjects? | **0** | Similar modeof datacollection were used |
| **9** | Were the **numerator(s) and denominator(s)** for the parameter of interest appropriate? | **0** | Yes,approperate parameters used |
|  | Overall risk of Bias | **4- moderate risk** |  |
